# Eculizumab discontinuation in a patient with atypical hemolytic uremic syndrome after ChAdOx1 nCoV-19 vaccination 

**DOI:** 10.5414/CNCS111070

**Published:** 2023-07-13

**Authors:** Marisa Roldão, Francisco Ferrer, Karina Lopes

**Affiliations:** Department of Nephrology, Centro Hospitalar do Médio Tejo, Torres Novas, Portugal

**Keywords:** atypical HUS, eculizumab, maintenance therapy discontinuation, ChAdOx1 nCoV-19 vaccine

## Abstract

Eculizumab has proven to be effective in patients with atypical hemolytic uremic syndrome (aHUS) in clinical trials and in the real world, but the optimal duration of therapy remains unknown. Standard maintenance treatment is often life-long, but the possibility of discontinuation has not yet been systematically tested. We describe a case of aHUS after ChAdOx1 nCoV-19 vaccination in a patient with homozygous *CFHR3/CFHR1* gene deletion who discontinued eculizumab maintenance therapy 24 weeks after achieving disease remission. We report the safety of discontinuing eculizumab treatment with the aim of minimizing the risk of adverse reactions, reducing the risk of meningitis, improving quality of life, and reducing the considerable treatment costs.

## Background 

Atypical hemolytic uremic syndrome (aHUS) is a life-threatening disease characterized by microangiopathic hemolytic anemia, thrombocytopenia, and kidney injury that results from thrombotic microangiopathy (TMA) within the glomerular capillaries and arterioles, involving a genetic or acquired dysregulation of the complement alternative pathway. A second trigger is often necessary for disease manifestation. Eculizumab is a humanized monoclonal complement inhibitor that binds with high affinity to C5, preventing the generation of the terminal complement complex C5b-9, thus inhibiting complement-mediated TMA. Eculizumab has proven to be effective in patients with aHUS in clinical trials [1], however, the optimal duration of therapy remains unknown. 

## Case report 

A 54-year-old female was admitted to the emergency department with general malaise, vomiting, and low urine output 5 days after receiving a first dose of ChAdOx1 nCoV-19 vaccine (Batch No. ABV3025; AstraZeneca, Cambridge, UK) [2]. Physical examination was unremarkable except for arterial hypertension (170/110 mmHg). Laboratory work-up revealed acute kidney injury (AKI), severe thrombocytopenia, and anemia, with elevated lactate dehydrogenase (LDH), undetectable haptoglobin, schistocytes on peripheral blood smear, and negative Coombs’s test. Urinalysis showed hemoglobinuria and proteinuria. Renal ultrasound revealed normal kidneys without hydronephrosis. Thrombotic microangiopathy was suspected, plasmapheresis (PEX) was immediately started, and a further work-up was performed, showing low C3 levels, normal activity of ADAMTS13, absence of Shiga toxin-producing *E. coli* in stool culture (including O157 strain), normal levels of factors H, B, and I, a normal activity of the alternate complement pathway, and absence of anti-factor H antibodies. Molecular genetic study did not recognize any pathogenic variants in the analyzed genes, but it identified a polymorphism described as a possible risk factor for aHUS development: deletion in homozygosity of *CFHR3/CFHR1*. She accomplished 10 sessions of PEX, with normalization of platelet count and anemia improvement. Considering anuric AKI and clinical signs of volume overload, hemodialysis was started after the second session of PEX. Given the absence of a secondary cause for TMA, a diagnosis of aHUS was made, and therapy with eculizumab was started. After the first administration, the patient resumed diuresis, renal function partially recovered, and hemodialysis was suspended. The patient was discharged with a hemoglobin level of 11.6 g/dL, normal platelet count, and creatinine 4.4 mg/dL. She maintained eculizumab therapy, 900 mg/week for 4 weeks, followed by 1,200 mg/every 2 weeks for 20 weeks with no evidence of hemolysis, normal platelet count, and improvement of renal function (creatinine 2 mg/dL), after which therapy with eculizumab was discontinued. She was then closely monitored with no evidence of relapse and stable renal function 24 weeks after treatment cessation. 

## Discussion 

Therapeutic terminal complement blockade with eculizumab changed the natural history of aHUS, resulting in a decreased risk of chronic kidney disease stage 5 from 60% to less than 15% [3]. At the same time, this advance raised several questions, mainly regarding the optimal duration of treatment, considering the risk of infections (meningitis), need for repeated infusions, and high cost. Few, small retrospective series have suggested that eculizumab discontinuation is safe and feasible in most patients, but that relapse risk appears to be higher in carriers of complement gene variants [3, 4]. Fakhouri et al. [4] performed the first prospective study of eculizumab discontinuation in 55 patients with aHUS who achieved remission. During follow-up, 13 patients relapsed. In multivariate analysis, female sex and the presence of a rare germline complement variant were associated with an increased risk of relapse. No patient without a germline complement mutation relapsed, suggesting that an eculizumab discontinuation strategy based on complement genetics is reasonable in aHUS patients [5]. In our case, genetic study did not identify any pathogenic variants in the 12 genes analyzed, but a homozygous *CFHR3/CFHR1* deletion, previously associated with aHUS and the production of anti-factor H antibodies, in the presence of a trigger, was found [2]. In fact, our patient did not relapse after 24 months of eculizumab discontinuation. Furthermore, in the study by Fakhouri et al. [4], all patients who relapsed recovered renal function after restarting eculizumab, suggesting the possibility of offering a trial of eculizumab discontinuation even in those with complement variants [5]. To our knowledge, new-onset, or relapse aHUS after COVID-19 vaccination has been previously described in 5 adult patients and 1 pediatric patient after the first, second, or even third dose of both mRNA and adenoviral-based COVID-19 vaccines [6, 7, 8, 9]. All patients had pathogenic complement variants and were treated with eculizumab. During eculizumab treatment, kidney function recovered in all but 1 patient. Eculizumab was discontinued in 2 patients after a median of 12 weeks, with no relapse [9]. Our case adds additional support to this hypothesis, but further studies are needed to clarify the best strategy for eculizumab discontinuation. 

## Patient consent 

Informed consent was obtained from the patient reported in this case. The participant has consented to the submission of the case report to the journal. 

## Funding 

No funding. 

## Conflict of interest 

The authors have no conflict of interest to declare that are relevant to the content of this article. 

## References

[1] Legendre CM Licht C Muus P Greenbaum LA Babu S Bedrosian C Bingham C Cohen DJ Delmas Y Douglas K Eitner F Feldkamp T Fouque D Furman RR Gaber O Herthelius M Hourmant M Karpman D Lebranchu Y Mariat C Terminal complement inhibitor eculizumab in atypical hemolytic-uremic syndrome. N Engl J Med. 2013; 368: 2169–2181. 23738544 10.1056/NEJMoa1208981

[2] Ferrer F Roldão M Figueiredo C Lopes K Atypical Hemolytic Uremic Syndrome after ChAdOx1 nCoV-19 Vaccination in a Patient with Homozygous CFHR3/CFHR1 Gene Deletion. Nephron. 2022; 146: 185–189. 34724668 10.1159/000519461 PMC8678224

[3] Brodsky RA Eculizumab and aHUS: to stop or not. Blood. 2021; 137: 2419–2420. 33956069 10.1182/blood.2020010234 PMC8109016

[4] Fakhouri F Fila M Hummel A Ribes D Sellier-Leclerc AL Ville S Pouteil-Noble C Coindre JP Le Quintrec M Rondeau E Boyer O Provôt F Djeddi D Hanf W Delmas Y Louillet F Lahoche A Favre G Châtelet V Launay EA Eculizumab discontinuation in children and adults with atypical hemolytic-uremic syndrome: a prospective multicenter study. Blood. 2021; 137: 2438–2449. 33270832 10.1182/blood.2020009280

[5] Ardissino G Testa S Possenti I Tel F Paglialonga F Salardi S Tedeschi S Belingheri M Cugno M Discontinuation of eculizumab maintenance treatment for atypical hemolytic uremic syndrome: a report of 10 cases. Am J Kidney Dis. 2014; 64: 633–637. 24656451 10.1053/j.ajkd.2014.01.434

[6] Rysava R Peiskerova M Tesar V Benes J Kment M Szilágyi Á Csuka D Prohászka Z Atypical hemolytic uremic syndrome triggered by mRNA vaccination against SARS-CoV-2: Case report. Front Immunol. 2022; 13: 1001366. 36275662 10.3389/fimmu.2022.1001366 PMC9580272

[7] Dos Santos C Chavarri A Alberto MF Fondevilla CG Sánched-Luceros A Recurrence of atypical hemolytic uremic syndrome after COVID-19 vaccination. Can J Kidney Health Dis. 2022; 9: 6.

[8] Claes KJ Geerts I Lemahieu W Wilmer A Kuypers DRJ Koshy P Ombelet S Atypical Hemolytic Uremic Syndrome Occurring After Receipt of mRNA-1273 COVID-19 Vaccine Booster: A Case Report. Am J Kidney Dis. 2023; 81: 364–367. 36342000 10.1053/j.ajkd.2022.07.012 PMC9484133

[9] Bouwmeester RN Bormans EMG Duineveld C van Zuilen AD van de Logt AE Wetzels JFM van de Kar NCAJ COVID-19 vaccination and Atypical hemolytic uremic syndrome. Front Immunol. 2022; 13: 1056153. 36531998 10.3389/fimmu.2022.1056153 PMC9755835

